# Pectus excavatum in adults over 40: a retrospective review of surgical experience

**DOI:** 10.1186/s13019-026-03890-8

**Published:** 2026-02-14

**Authors:** Çağatay Çetinkaya, Nezih Onur Ermerak, Mustafa Yüksel

**Affiliations:** 1 Department of Thoracic Surgery, Uskudar University School of Medicine, Istanbul, Turkey; 2 Department of Thoracic Surgery, Marmara University School of Medicine, Istanbul, Turkey; 3 Department of Thoracic Surgery, Bilim University School of Medicine, Istanbul, Turkey

**Keywords:** Pectus excavatum, Nuss procedure, Adults, Chest wall deformity, Surgical outcomes

## Abstract

**Background:**

Pectus excavatum is the most common congenital anterior chest wall deformity, typically corrected during adolescence. However, an increasing number of adult patients seek surgical repair due to persistent symptoms or cosmetic concerns. Surgical correction in adults poses unique challenges, and data on outcomes in older patients remain limited. This study aimed to evaluate the clinical characteristics, surgical techniques, and postoperative outcomes of pectus excavatum repair in patients aged 40 years and older.

**Methods:**

We retrospectively analyzed adult patients aged ≥ 40 who underwent surgical repair for pectus excavatum at our center between 2012 and 2025. Clinical indications, operative details, and postoperative outcomes were evaluated. Patients younger than 40 years were used as a comparative cohort. Postoperative complications were categorized as minor or major according to the Clavien-Dindo classification system.

**Results:**

A total of 16 patients (mean age: 46.9 years) underwent minimally invasive repair of pectus excavatum (MIRPE). A cross-bar configuration was applied in 56.3% of patients, and two or more bars were required in 87.5%. The most common surgical indication was symptomatic presentation (50%). Postoperative complications occurred in 5 patients (31.3%), including 3 major and 2 minor events. Compared with younger patients (*n* = 1180), the older cohort demonstrated a higher complication rate, although this difference did not reach statistical significance.

**Conclusion:**

Surgical repair of pectus excavatum in patients over the age of 40 is feasible and safe when appropriate techniques are applied. Despite increased anatomical stiffness necessitating complex configurations, acceptable clinical outcomes can be achieved. Age alone should not be considered a contraindication for pectus repair in well-selected adult patients.

**Supplementary Information:**

The online version contains supplementary material available at 10.1186/s13019-026-03890-8.

## Introduction

 Pectus excavatum (PE) is the most common congenital anterior chest wall deformity, with an estimated prevalence of 0.3% among live births [1]. Although the condition is typically diagnosed and treated during adolescence, recent studies have identified its persistence into adulthood, with a reported adult prevalence of 0.4% based on population-level imaging data [2].

The etiology of PE remains uncertain despite various hypotheses, and the deformity predominantly affects males, with a reported male-to-female ratio of approximately 5:1 [3].

Surgical correction of PE has evolved significantly. Ravitch sternoplasty, first described by Mark M. Ravitch in 1949, was initially the gold-standard open technique [4]. In 1998, Dr. Donald Nuss introduced the minimally invasive Nuss procedure—a method relying on subtsternal bar placement without cartilage resection—which rapidly gained popularity, especially among pediatric and adolescent patients [5]. Since then, additional modifications and technical refinements have been developed to address anatomical variations and older patient cohorts.

Surgical repair of PE is typically performed during adolescence, with multiple reports indicating that the optimal timing lies between 12 and 16 years of age, when chest wall flexibility is maximal and recurrence is minimized [6, 7]. While adult repair is feasible, procedural complexity increases with age due to reduced chest wall pliability and potential comorbidities [3, 8]. Nonetheless, recent large institutional cohorts have demonstrated that the minimally invasive Nuss procedure can be performed safely and effectively in adult patients, with favorable cosmetic outcomes and high patient satisfaction [9].

In this study, we retrospectively analyzed a cohort of patients aged 40 years and older who underwent surgical correction of pectus excavatum. Our aim is to evaluate the surgical indications, techniques, and outcomes in this underrepresented group. By doing so, we hope to contribute to a better understanding of how patient age may influence clinical decision-making in pectus surgery.

## Patients and Methods

Our center performed surgical repair of pectus excavatum in 1196 patients between May 2005 and June 2025. From this cohort, we retrospectively analyzed patients aged 40 years and older who underwent surgical correction between May 2012 and June 2025. All procedures were performed by the same thoracic surgery team.

Surgical technique was selected based on deformity type and patient characteristics. Although both minimally invasive repair (Nuss procedure) and open modified Ravitch procedure were performed at our center, only patients who underwent the Nuss procedure were included in this study. The number of pectus bars and the use of cross-bar configuration was determined intraoperatively according to anatomical requirements. The indications and technical aspects of the cross-bar configuration have been previously described in detail in our earlier publication [10].

All relevant data including patient demographics, operative details, length of hospital stay, and postoperative follow-up (such as bar removal and the need for revision surgery) were retrospectively collected from patient records. Preoperative computed tomography was performed selectively in patients with clinically severe deformity, suspected cardiac compression, or symptom-driven presentation. Transthoracic echocardiography was obtained in selected patients with symptomatic presentation or suspected cardiac compression to evaluate potential cardiac involvement. Available preoperative Haller index values were recorded for objective anatomical assessment.

In addition to analyzing patients aged 40 and above, we conducted a comparative evaluation using data from the remaining 1180 patients under 40 years of age who underwent pectus excavatum repair during the same study period. Bar number (including use of 1, 2, 3, or more bars), application of cross-bar technique, and surgical indication (cosmetic vs. symptomatic) were compared between the two age groups. Statistical analysis was performed to assess the significance of differences in these variables, with a p-value < 0.05 considered significant.

Postoperative complications were identified and classified retrospectively for both the ≥ 40 and < 40 age groups. Complications were stratified using the Clavien-Dindo classification system, which provides a standardized grading of surgical complications based on the level of intervention required. Minor complications were defined as Grade I–II events, such as self-limited issues or those requiring pharmacologic treatment only, while major complications included Grade III or higher events necessitating surgical, endoscopic, or radiologic intervention. This classification enabled consistent comparison of complication profiles across age groups [11].

A complete photographic archive, including preoperative and postoperative images, was maintained for all patients. Postoperative follow-up was routinely scheduled at 1 month and 6 months after surgery, and every 6 months thereafter until bar removal. This study was approved by the Institutional Ethics Committee of our institution.

## Results

A total of 16 patients (12 males and 4 females) aged 40 years and older underwent surgical correction for pectus excavatum during the study period. The mean age was 46.9 ± 6.6 years (range: 40–58). Chest wall deformities were classified as symmetric in 9 patients and asymmetric in 7 patients. None of the patients had a prior history of pectus surgery or relevant comorbidities. Preoperative computed tomography was available in 10 patients, in whom the mean Haller index was 4.7 ± 1.2 (range: 3.1–7.1), reflecting moderate to severe chest wall deformity. The demographic and preoperative characteristics of the patients are summarized in Table 1. Detailed individual patient characteristics and outcomes are summarized in Supplementary Table 1.

**Table 1:** Patient demographics and preoperative characteristics

Parameter	Value
Number of the patients (n)	16
Age (year)	46.9 ± 6.6 (40–58)
Sex (n)	
Female	4 (25%)
Male	12 (75%)
Type of deformity (n)	
Symmetrical	9 (56.3%)
Asymmetrical	7 (43.7%)
Comorbidities	None
Past surgical history	None
Indication for surgery (n)	
Cosmetic concern	8 (50%)
Exertional dyspnea	3 (18.8%)
Preventive	2 (12.5%)
Postprandial tachycardia	1 (6.3%)
Orthostatic symptoms	1 (6.3%)
Chest discomfort	1 (6.3%)

Of the 16 patients, 8 (50%) underwent surgery primarily due to cosmetic concerns, while 8 (50%) reported symptomatic indications. These included exertional dyspnea (*n* = 3), postprandial tachycardia (*n* = 1), and chest discomfort and palpitations (*n* = 1). Additionally, 3 patients underwent preventive surgery due to suspected cardiac compression or orthostatic symptoms. Indications for surgery are listed in Table 1. Preoperative transthoracic echocardiography was performed in 11 patients with symptomatic presentation or clinically suspected cardiac compression. Cardiac compression was identified in 5 patients, all of whom underwent repeat postoperative echocardiographic evaluation, demonstrating complete resolution of cardiac compression following surgical repair. Among the 8 symptomatic patients, 6 reported complete resolution of symptoms during postoperative follow-up.

Minimally invasive repair using the Nuss procedure was performed in all 16 patients. One bar was placed in 2 patients, two bars in 8 patients, and three bars in 6 patients. The cross-bar technique was applied in 9 patients. Intraoperative and postoperative findings, including bar configuration and hospital course, are detailed in Table 2.

**Table 2:** Intraoperative and postoperative details

Parameter	Value
Mean duration of surgery (min)	88.8 ± 23.6 (60–120)
Mean length of hospital stay (days)	5.2 ± 1.1 (4–8)
Number of bars (n)	
1 bar	2 (12.5%)
2 bars	8 (50%)
3 bars	6 (37.5%)
Bar configuration (n)	
1 bar	2 (12.5%)
Parallel bar	5 (31.5%)
Cross bar	9 (56.3%)
Bar removal (n)	
Removed	11 (68.7%)
Pending removal	5 (31.5%)

**Table 3:** Comparison of surgical details by age group

Parameter	Age ≥ 40 (*n* = 16)	Age < 40 (*n* = 1180)	p-value
Bar count			
o 1 bar	2 (12.5%)	597 (50.5%)	
o 2 bar	8 (50%)	504 (42.7%)	
o 3 bar	6 (37.5%)	76 (6.5%)	
o 4 bar	0 (0%)	3 (0.3%)	
Patients receiving ≥ 2 bars	14 (77.8%)	583 (49.4%)	**p = 0.002**
Cross-bar configuration	9 (56.3%)	152 (12.9%)	**p < 0.001**
Indications for surgery			
o Cosmetic concern	8 (50.0%)	59 (5.0%)	**p < 0.001**
o Symptomatic (e.g. dyspnea, chest pain)	8 (50.0%)	1121 (95%)	
Postoperative complications			
o Minor complications	2 (12.5%)	77 (6.5%)	p = 0.36
o Major complications	3 (18.8%)	60 (5.1%)	**p = 0.018**

When compared to patients under 40 years of age (*n* = 1180), those aged 40 years and older (*n* = 16) were significantly more likely to receive two or more pectus bars (87.5% vs. 49.4%, *p* = 0.002) and to undergo surgery using a cross-bar configuration (56.3% vs. 12.9%, *p* < 0.001). Accordingly, more complex bar configurations were more frequently used in the older age group (Table 3).

Additionally, a significant difference was observed in the primary indications for surgery. Among patients under 40 years of age, 59 of 1180 (5.0%) underwent surgery for symptomatic reasons, while 1121 (95.0%) reported cosmetic concerns. In contrast, the distribution was equal in patients aged 40 years and older, with 8 (50.0%) undergoing surgery for cosmetic reasons and 8 (50.0%) for symptomatic indications. This difference was statistically significant (*p* < 0.001).

The mean operation time was 88.8 ± 23.6 min, and the mean length of hospital stay was 5.2 ± 1.1 days.

Postoperative complications occurred in 5 of 16 patients aged 40 years and older (31.3%), including 3 major and 2 minor complications. These included pleural effusion requiring chest tube drainage (*n* = 1), early bar removal due to severe pain (*n* = 2), self-limiting atelectasis (*n* = 1), and superficial wound infection managed conservatively (*n* = 1). In comparison, among 1180 patients under 40 years of age, complications were observed in 137 patients (11.6%), including 60 major and 77 minor complications. The most common complications in the entire cohort were wound-related issues (*n* = 42), including infection, granulation, or seroma, of which 23 required surgical revision. Pleural space complications (pneumothorax or effusion) were identified in 18 patients, with 9 requiring drainage procedures. Additionally, bar revision was performed in 9 patients due to rotation or inadequate correction, while early bar removal was required in 6 patients due to pain or allergic reactions.

When postoperative complications were analyzed, patients aged 40 years and older demonstrated a higher overall complication rate compared with younger patients (31.3% vs. 11.6%, *p* = 0.017). The incidence of major complications was also significantly higher in the older group (18.8% vs. 5.1%, *p* = 0.018). In contrast, minor complication rates did not differ significantly between the two age groups (12.5% vs. 6.5%, *p* = 0.36).

All patients received standardized analgesia, including morphine-based intravenous patient-controlled analgesia (PCA) during the first two postoperative days. Following PCA discontinuation, a combination of intravenous tramadol and paracetamol was administered until discharge. After discharge, oral analgesics including NSAIDs, opioids, and paracetamol were prescribed as needed.

In total, bars were removed in 11 patients, with the time between placement and removal ranging from 5 to 41 months.

**Fig. 1:**
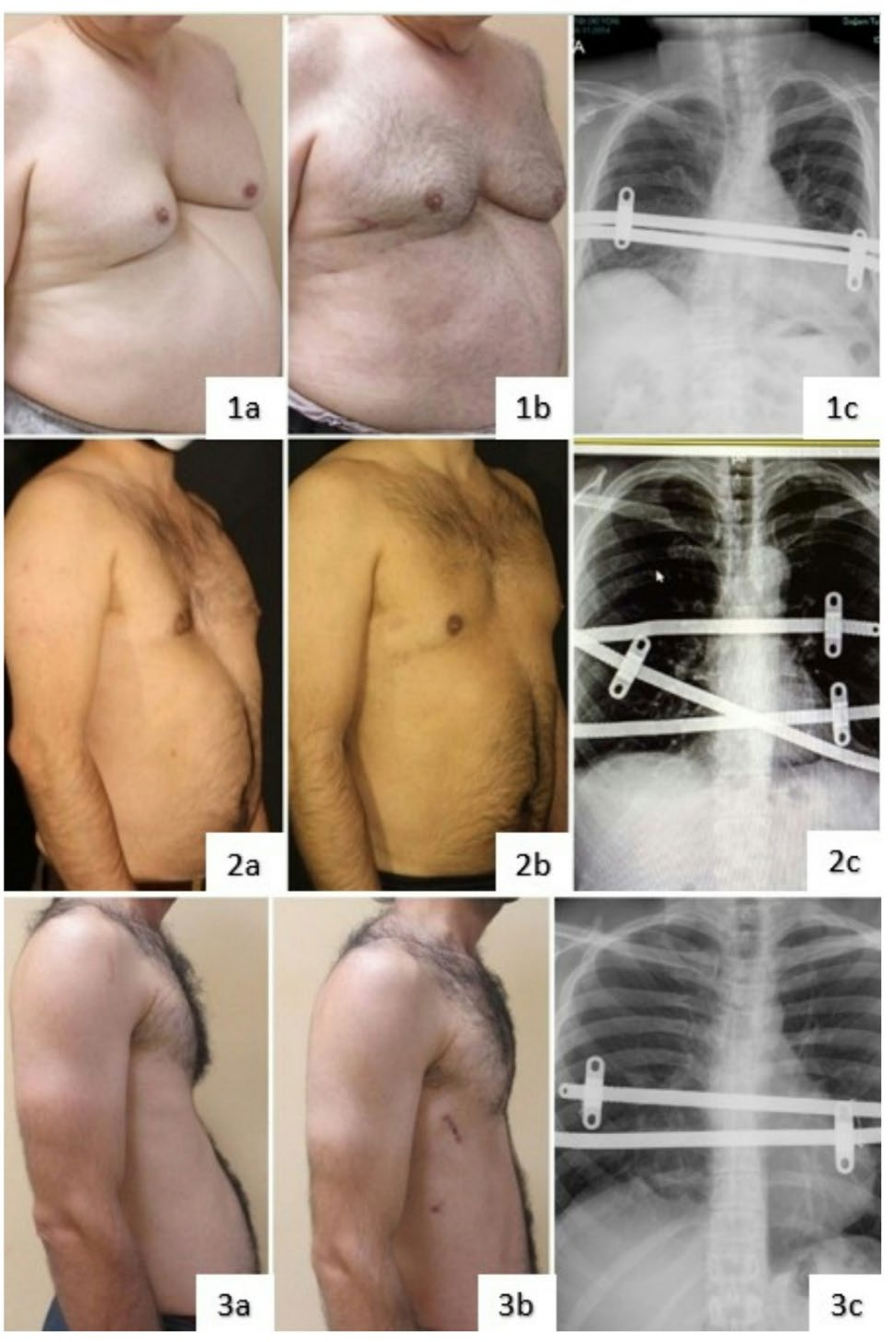
Representative preoperative and postoperative clinical photographs and chest radiographs of adult patients aged over 40 years who underwent minimally invasive repair of pectus excavatum using the Nuss procedure (**1a**–**1c**) A patient treated with two parallel bars (**2a**–**2c**) A patient treated with three bars using a cross-bar configuration (**3a**–**3c**) A patient with deep asymmetric pectus excavatum treated with two parallel bars

Representative preoperative and postoperative clinical images of adult patients treated with minimally invasive repair are shown in Fig. 1. These images illustrate different bar configurations and deformity types following minimally invasive repair.

## Discussion

Pectus excavatum is most commonly corrected during adolescence, when the chest wall remains flexible and surgical outcomes are generally favorable [12]. Minimally invasive repair using the Nuss procedure has become the standard approach, particularly in younger patients, due to its cosmetic effectiveness and relatively low complication rates [5]. However, surgical correction is not limited to pediatric populations. In selected adult patients, especially those with significant symptoms or functional limitations, surgical intervention may still offer substantial benefit.

Surgical repair in older adults presents distinct challenges compared to younger patients. Adults frequently exhibit increased chest wall rigidity, resulting in prolonged operative times and heightened technical difficulty in bar placement. Moreover, higher rates of perioperative complications—such as bar displacement, pleural effusion, and extended postoperative pain—have been well documented in this population. For instance, de Loos et al. found a significantly greater incidence of complications in adults compared to younger cohorts undergoing the Nuss procedure [13]. Similarly, Rshaidat et al., in a national database analysis, reported increased complication rates among adult patients, emphasizing the importance of careful risk–benefit assessment [14]. These findings are consistent with our current series, where advanced chest wall rigidity in older patients necessitated more complex bar configurations. Specifically, two or more bars were used in 14 out of 16 patients (87.5% vs. 49.4% in younger patients, *p* = 0.002), and the cross-bar technique was applied in 9 patients (56.3% vs. 12.9%, *p* < 0.001) to achieve adequate correction. These differences were statistically significant and align with previous reports highlighting the technical challenges and increased support requirements in adult pectus repair [13, 14]. In our series, the presence of preoperative cardiac compression on echocardiography and its complete postoperative resolution in affected patients support a clinically meaningful association between chest wall deformity and cardiopulmonary symptoms in selected adult patients. Postoperative anatomical assessment was primarily based on clinical and radiographic follow-up rather than routine cross-sectional imaging. Routine postoperative computed tomography was therefore not performed in our practice, as radiologic follow-up after pectus repair should be balanced against unnecessary radiation exposure, particularly in the absence of clinical indication.

A comparative study by Hasan et al. investigated surgical outcomes in adult pectus excavatum patients by analyzing two age groups—those above and below 40 years of age—following minimally invasive repair. Their report highlighted factors such as symptom profiles, bar usage, and selected perioperative outcomes in a small cohort [15]. In our current series, a similar age-based classification was applied; however, additional factors such as bar configuration complexity, complication rates, and long-term follow-up were also incorporated, enabling a more detailed evaluation of surgical outcomes in older adults.

In parallel with the documented technical challenges, several studies have demonstrated that the Nuss procedure can still be performed safely and effectively in appropriately selected adult patients. Mack et al. reported favorable outcomes in adults across high-volume centers, with complication rates comparable to those seen in younger populations when appropriate surgical expertise and perioperative protocols were in place [16]. Similarly, de Loos et al. emphasized that, despite an increased risk profile in adults, the overall complication rates remained acceptable, supporting the use of minimally invasive techniques in older individuals [13]. Our results align with previously published data: although postoperative complications occurred in 31.3% of patients over 40 years of age, including both minor and major events, no life-threatening complications were observed, and no patient required revision surgery during follow-up. Although bar removal is conventionally planned after a longer implantation period, early bar removal may be required in selected cases due to severe pain or intolerance; in our series, the shortest bar retention time reflected complication-driven management, including complete bar removal in one patient and partial (single-bar) removal in another. These outcomes suggest that, in experienced hands, surgical repair of pectus deformities in adults over 40 can be performed safely with an acceptable rate of complications.

In adult patients, surgical indications often extend beyond cosmetic concerns and may reflect genuine functional impairment. Comprehensive reviews recommend considering corrective surgery when symptoms such as exertional dyspnea, chest pain, palpitations, or signs of cardiopulmonary compromise are present, rather than relying solely on anatomical severity.

In our cohort, 50.0% of patients underwent surgery due to symptomatic indications such as exercise intolerance or presyncopal episodes, while the remaining 50.0% underwent repair for cosmetic concerns alone. In contrast, only 5.0% of patients under the age of 40 underwent surgery for symptomatic reasons, indicating a statistically significant difference between age groups (*p* < 0.001). These results are consistent with previous studies highlighting the predominance of physiologic complaints in older patients seeking pectus repair. For instance, Jaroszewski et al. reported that adult patients undergoing minimally invasive repair experienced significant relief of symptoms, including improved exercise capacity and decreased cardiopulmonary limitations [17].

These collective findings suggest that, beyond cosmetic benefits, surgical correction of pectus excavatum in older adults can address clinically significant symptoms and improve overall quality of life. Our current series further supports this perspective by demonstrating a high rate of symptom-driven indications in the over-40 population.

These findings should be interpreted in the context of several limitations inherent to the study design. Due to the retrospective nature of the study, not all patients underwent uniform preoperative imaging or functional assessment. Although pulmonary function tests were routinely performed in clinical practice, complete and analyzable pulmonary function data were not available for all patients and therefore could not be systematically evaluated. In addition, postoperative computed tomography was not routinely obtained, limiting objective pre–post anatomical comparisons based on imaging. Body habitus and body mass index were not used as matching variables between age groups, which may have influenced surgical complexity and postoperative outcomes and should be considered when interpreting intergroup comparisons. Finally, the relatively small number of patients aged 40 years and older reflects the selective nature of surgical referral in this population and may limit the generalizability of the results.

## Conclusion

Our findings demonstrate that surgical correction of pectus excavatum in patients aged 40 years and older can be performed safely and effectively, with an acceptable complication rate. Older adults frequently required more complex bar configurations, reflecting the technical challenges associated with reduced chest wall compliance. Furthermore, the high proportion of symptom-driven indications underscores the clinical relevance of the deformity in this age group. Overall, our results support the notion that age alone should not be considered a contraindication for surgical repair, and that appropriately selected adult patients may achieve meaningful functional and symptomatic improvement.

## Supplementary Information

Supplementary Material 1

## Data Availability

No datasets were generated or analysed during the current study.

## Abbreviations

PE: Pectus excavatum
MIRPE: Minimally invasive repair of pectus excavatum
PCA: Patient-controlled analgesia
CT: Computed tomography
NSAIDs: Non-steroidal anti-inflammatory drugs
IV: Intravenous
n: Number of patients
