# Supplementary material for: Pectus excavatum in adults over 40: a retrospective review of surgical experience
Source: J Cardiothorac Surg. 2026 Feb 14;21:106. doi: 10.1186/s13019-026-03890-8 (PMC12951984; doi:10.1186/s13019-026-03890-8)
Supplement: Supplementary file 1 — Supplementary Material 1 [file 13019_2026_3890_MOESM1_ESM.docx]

| \| **Patient** \| \| --- \|  \|  \| \| --- \|  \|  \| \| --- \|  \|  \| \| --- \|  \|  \| \| --- \|  \|  \| \| --- \| | \| **Age** \| \| --- \| | \| **Sex** \| \| --- \| | **Deformity (S/A)** | **Height / Weight** | \| **Preop HI** \| \| --- \| | **Preop ECHO (Compression)** | \| **Number**  **of bars** \| \| --- \| | \| **Cross-bar** \| \| --- \| | \| **Complication** \| \| --- \| | **Bar Removal**  **(months)** | **Duration of surgery**  **(minutes)** | \| **Follow-up (months)** \| \| --- \| |
| --- | --- | --- | --- | --- | --- | --- | --- | --- | --- | --- | --- | --- | --- | --- | --- | --- | --- | --- | --- | --- | --- | --- | --- | --- | --- |
| 1 | 46 | M | S | 170/74 | NA | NA | 1 | No | None | 38 | 60 | 163 |
| 2 | 46 | M | S | 172/80 | 5.4 | Yes | 2 | No | None | 41 | 120 | 145 |
| 3 | 58 | M | S | 168/71 | 4.5 | No | 2 | No | None | 38 | 120 | 139 |
| 4 | 56 | F | S | 155/60 | 6.2 | Yes | 2 | No | None | 37 | 60 | 137 |
| 5 | 40 | M | A | 180/85 | 3.6 | No | 2 | No | None | 37 | 120 | 135 |
| 6 | 49 | F | A | 160/65 | NA | NA | 1 | No | None | 48 | 60 | 92 |
| 7 | 56 | M | A | 165/75 | 4.2 | Yes | 3 | Yes | Wound infection | 40 | 120 | 78 |
| 8 | 55 | M | A | 172/81 | 4.6 | No | 3 | Yes | None | 38 | 100 | 66 |
| 9 | 42 | F | A | 168/56 | NA | NA | 3 | Yes | None | 32 | 100 | 44 |
| 10 | 41 | F | S | 162/58 | 7.1 | Yes | 2 | Yes | Early removal of 1 bar due to pain | Pending | 90 | 40 |
| 11 | 41 | M | S | 176/79 | 3.8 | NA | 3 | Yes | None | 35 | 90 | 38 |
| 12 | 51 | M | S | 169/72 | 3.1 | No | 2 | Yes | Pleural effusion | Pending | 90 | 38 |
| 13 | 40 | M | S | 174/75 | NA | No | 2 | Yes | Early bar removal due to pain | 5 | 80 | 20 |
| 14 | 40 | M | A | 182/83 | 4.5 | Yes | 3 | Yes | None | Pending | 90 | 17 |
| 15 | 40 | M | A | 171/73 | NA | No | 2 | No | Atelectasis | Pending | 60 | 9 |
| 16 | 40 | M | S | 172/78 | NA | NA | 3 | Yes | None | Pending | 60 | 6 |

**Supplementary Table 1. Individual Demographic, Operative, and Follow-up Characteristics of Patients Aged ≥40 Years**

Abbreviations:

NA: Not available

Pending: Pending indicates that bar removal had not yet been performed at the time of last follow-up.

HI: Haller Index

S: Symmetric

A: Asymmetric
